# Li_0.17_Na_5.83_Mo_11_O_36_

**DOI:** 10.1107/S1600536812044224

**Published:** 2012-11-03

**Authors:** Ines Ennajeh, Hamadi Hamza, Mohamed Faouzi Zid, Ahmed Driss

**Affiliations:** a Laboratoire de Matériaux et Cristallochimie, Faculté des Sciences de Tunis, Université de Tunis ElManar, 2092 Manar II Tunis, Tunisia

## Abstract

The title mixed-alkali-metal molybdenium oxide, hexa­kis­(lithium/sodium) undeca­molybdate, was synthesized by solid-state reaction at 793 K. Its [Mo_11_O_36_]^6−^ framework is built up from MoO_6_ octa­hedra and MoO_5_ pyramids linked together by edges and vertices. The framework delimits two types of inter­secting tunnels running along [100] and [001], where the sodium and lithium ions are located. Two of the sodium ions and the lithium ion have fractional site occupancies. One of the Mo atoms has site symmetry 2, one sodium ion has site symmetry 2 and one has site symmetry -1, and the Li^+^ ion has site symmetry 2. Structural relationships between the title compound and the anatase and Na_6_Mo_11_O_36_ structures are discussed.

## Related literature

For complex oxides containing lithium ions, see: Daidouh *et al.* (1997 ▶); Whittingham & Silbernagel (1976 ▶); Mizushima *et al.* (1980 ▶); Kanno *et al.* (1994 ▶); Guilmard *et al.* (2003 ▶); Lin *et al.* (2005 ▶); Yuh *et al.* (1995 ▶); Koyama *et al.* (2004 ▶). For details of structurally related compounds, see: Bramnik & Ehrenberg (2004 ▶); Caillet (1967 ▶); Seleborg (1967 ▶). For further synthetic details, see: Bramnik & Ehrenberg (2004 ▶). For bond-valence sums, see: Brown & Altermatt (1985 ▶). For related literature, see: Koyama *et al.* (2004 ▶).

## Experimental

### 

#### Crystal data

Li_0.17_Na_5.83_Mo_11_O_36_
*M* *_r_* = 1766.55
Monoclinic, 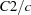

*a* = 7.2250 (9) Å
*b* = 17.863 (2) Å
*c* = 22.086 (3) Å
β = 90.162 (8)°
*V* = 2850.5 (6) Å^3^
*Z* = 4
Mo *K*α radiation
μ = 4.89 mm^−1^
*T* = 298 K
0.30 × 0.24 × 0.14 mm

#### Data collection

Enraf–Nonius CAD-4 diffractometer
Absorption correction: ψ scan (North *et al.*, 1968 ▶) *T*_min_ = 0.25, *T*_max_ = 0.50
6662 measured reflections
3138 independent reflections
3065 reflections with *I* > 2σ(*I*)
*R*_int_ = 0.032
2 standard reflections every 120 min intensity decay: 2.3%

#### Refinement

*R*[*F* ^2^ > 2σ(*F* ^2^)] = 0.037
*wR*(*F* ^2^) = 0.095
*S* = 1.33
3138 reflections
247 parameters
1 restraint
Δρ_max_ = 1.39 e Å^−3^
Δρ_min_ = −1.85 e Å^−3^

### 

Data collection: *CAD-4 EXPRESS* (Duisenberg, 1992 ▶; Macíček & Yordanov, 1992 ▶); cell refinement: *CAD-4 EXPRESS*; data reduction: *XCAD4* (Harms & Wocadlo, 1995 ▶); program(s) used to solve structure: *SHELXS97* (Sheldrick, 2008 ▶); program(s) used to refine structure: *SHELXL97* (Sheldrick, 2008 ▶); molecular graphics: *DIAMOND* (Brandenburg, 1998 ▶); software used to prepare material for publication: *WinGX* (Farrugia, 1999 ▶).

## Supplementary Material

Crystal structure: contains datablock(s) I, global. DOI: 10.1107/S1600536812044224/hb6937sup1.cif

Structure factors: contains datablock(s) I. DOI: 10.1107/S1600536812044224/hb6937Isup2.hkl

Additional supplementary materials:  crystallographic information; 3D view; checkCIF report

## Figures and Tables

**Fig. 1.**
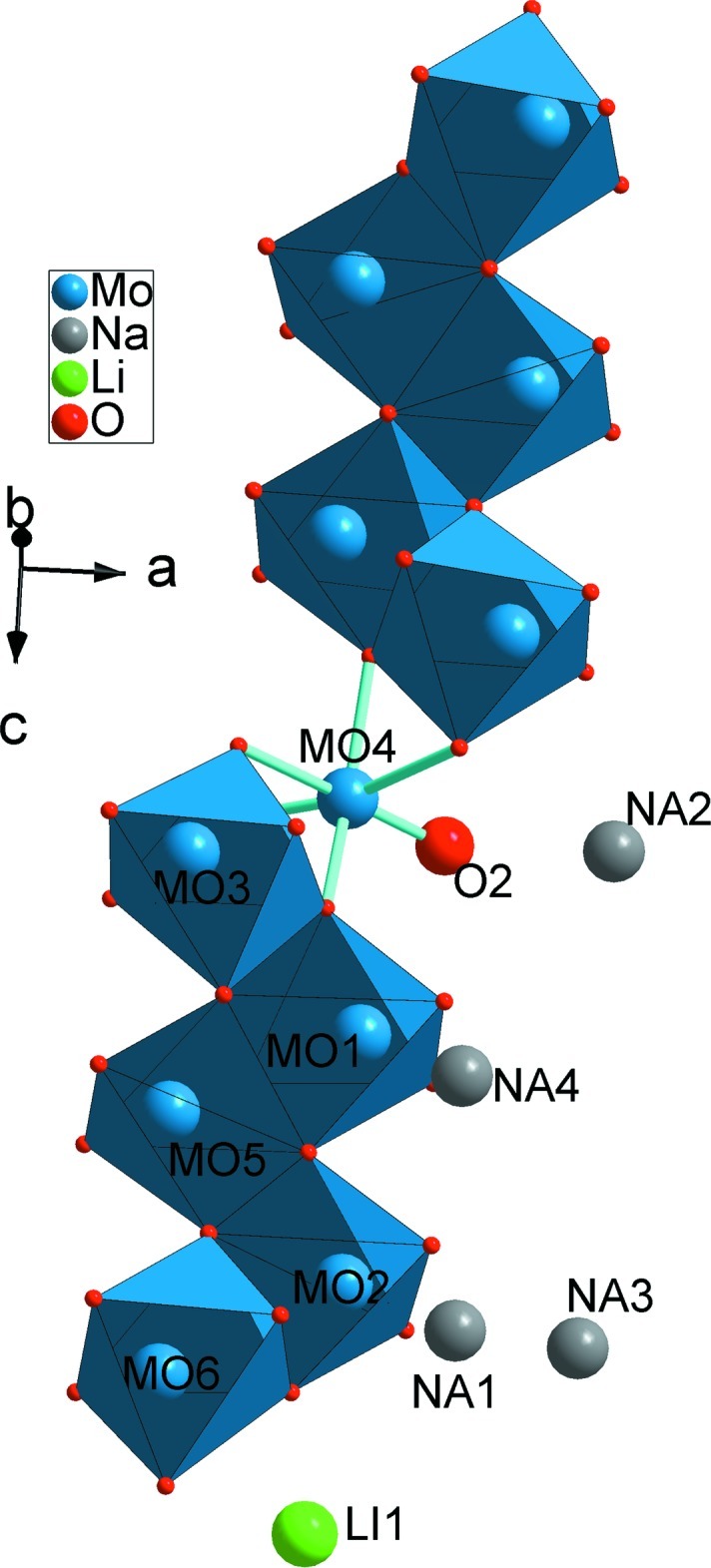
Unité asymétrique dans Li0.17Na5.83Mo11O36.

**Fig. 2.**
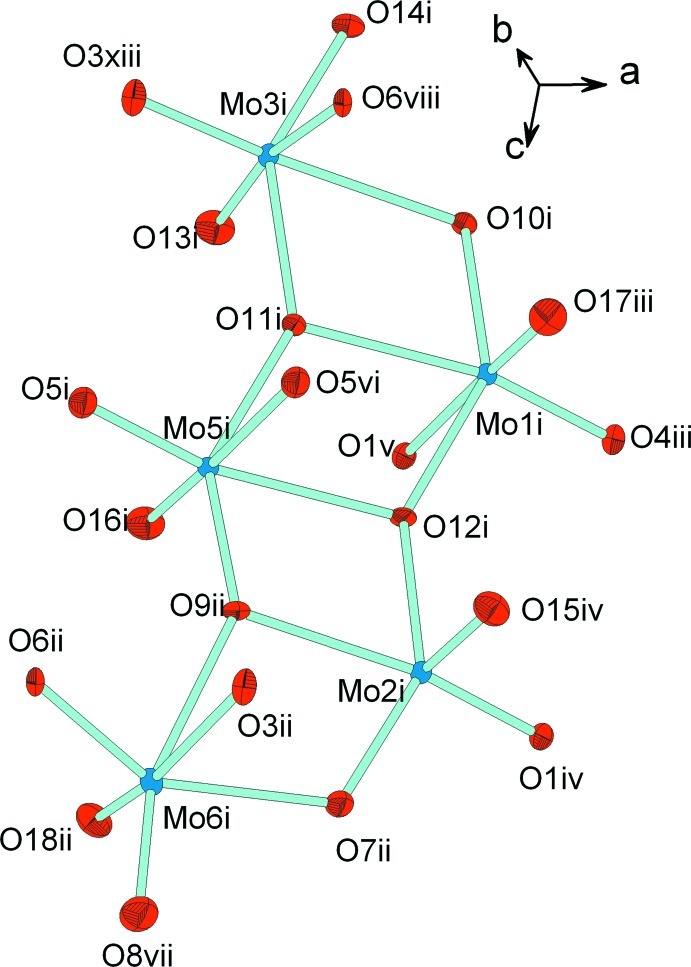
Cluster Mo5O21 dans Li0.17Na5.83Mo11O36. Les éllipsoïdes ont été définis avec 50% de probabilité. [Code de symétrie: (i) 1/2 - x, 1/2 + y,1/2 - z; (ii) 1/2 - x, 1/2 - y, 3/2 - z; (iii) 3/2 - x, 1/2 + y, 1/2 - z; (iv) 3/2 - x, 1/2 + y, 3/2 - z; (v) x - 1/2, 1/2 - y, z - 1/2; (vi) x, 1 - y, 1/2 - z; (vii) x - 1/2, 1/2 + y, z; (viii) x, 1 - y, z - 1/2].

**Fig. 3.**
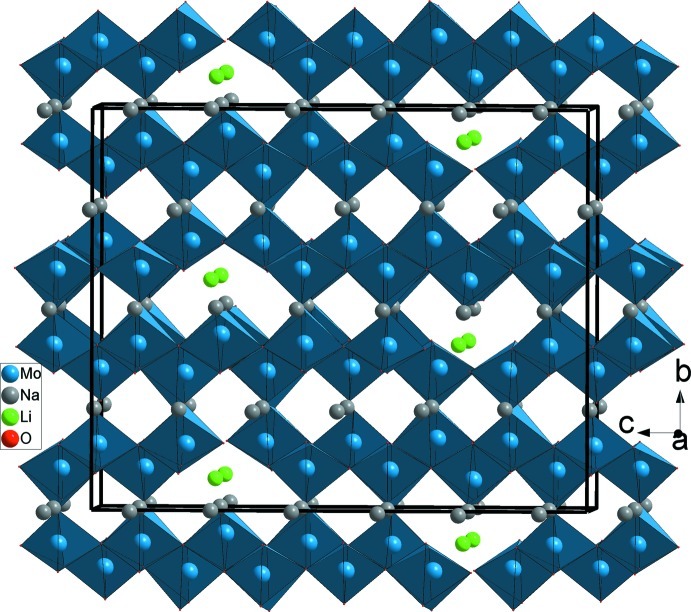
Projection de la charpente anionique selon a montrant la jonction des unités Mo11O36 dans la structure.

**Fig. 4.**
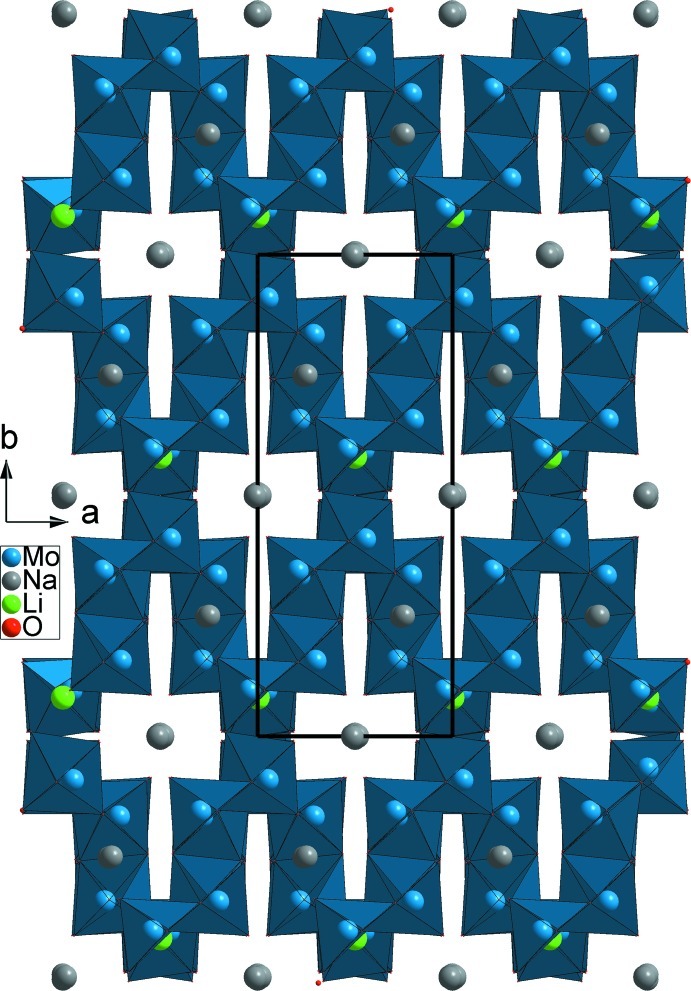
Projection de la structure de Li0.17Na5.83Mo11O36, selon c, mettant en évidence les canaux où logent les cations Na+.

**Fig. 5.**
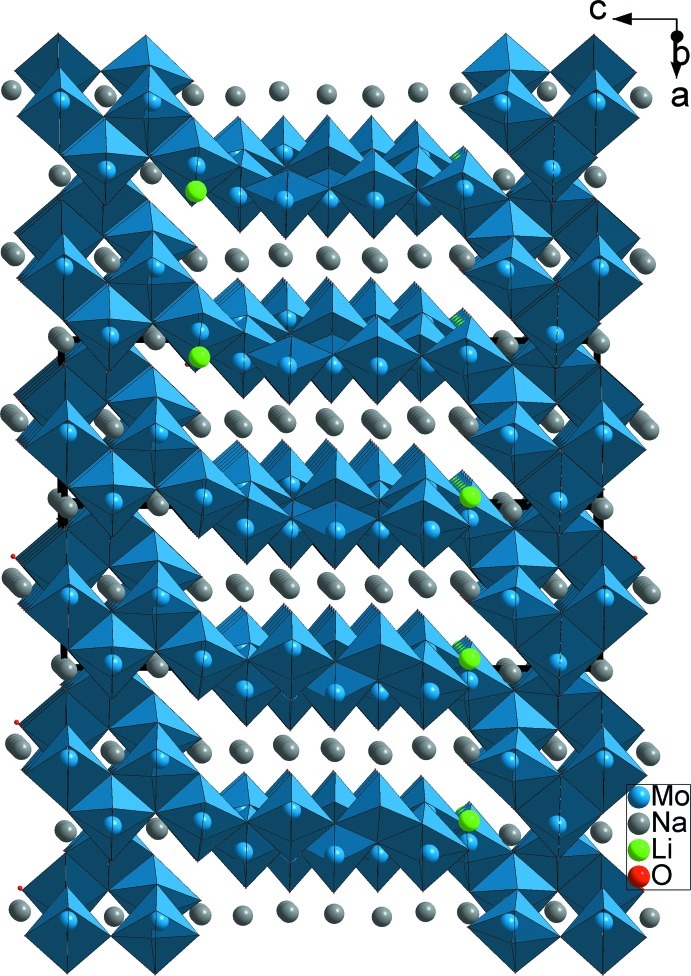
Projection de la structure de Li0.17Na5.83Mo11O36, selon la direction [-110], montrant des canaux allongés où résident les cations Na+.

## supplementary crystallographic information

### Comment

Ces dernières années, un grand intérêt a été accordé à l'étude des conducteurs ioniques pour l'importance de leur application dans le domaine de l'énergétique et de l'électronique, en particulier, les conducteurs ioniques où le porteur de charge est un cation monovalent (Li, Na, Ag) (Daidouh *et al.*, 1997). Les conducteurs par l'ion lithium telques Les matériaux LiMO_2_ (*M*= Mn, Fe, Co, Ni) (Whittingham & Silbernagel, 1976; Mizushima *et al.*, 1980; Kanno *et al.*, 1994) et les matériaux substitués telques Li*M_y_*Co_1 -_*_y_*O_2_ et Li*M_y_*Ni_1 -_*_y_*O_2_ (Koyama *et al.*, 2004; Guilmard *et al.*, 2003; Lin *et al.*, 2005) ont pris un grand intérêt dans la réalization des générateurs électrochimiques de haute densité d'énergie. De plus, La découverte des batteries de type Li-ion rechargeable telques les batteries à base de LiCoO_2_ (Yuh *et al.*, 1995) a encouragé la recherche dans cet axe, en raison de leur forte densité énergétique, faible coût des matières premières et respect de l'environnement et de sécurité. Plusieurs équipes visent à améliorer ces batteries et de créer des générations nouvelles par substitution dans ces composés. Dans ce cadre, on a essayé d'une part, d'explorer le système Li_2_O–Na_2_O–MoO_3_ et d'autre part d'augmenter la mobilité des ions monovalents dans les composés rencontrés dans la littérature Na_6_Mo_11_O_36_ (Bramnik & Ehrenberg, 2004), Na_2_Mo_3_O_10_ et Na_2_Mo_5_O_16_ (Caillet, 1967), Na_2_Mo_2_O_7_ (Seleborg, 1967) en substituant l'ion sodium par le lithium de taille plus faible. Ceci nous a conduit à la synthèse, par réaction à l'état solide, d'un nouveau molybdenium oxyde double de sodium et de lithium de formulation Li_0.17_Na_5.83_Mo_11_O_36_. *L*'unité *asym*étrique est construite par deux groupements identiques Mo_5_O_21_ reliés par mize en comment d'arêtes à un octaèdre Mo4O_6_ (Fig. 1). Dans ces derniers clusters Mo_5_O_21_ quatre octaèdres MoO_6_ se connectent au moyen d'une pyramide Mo2O_5_ (Fig. 2). En effet, dans la charpente anionique chaque unité structurale Mo_11_O_36_ se lie à quatre identiques par partage d'arêtes ou bien de sommets (Fig. 3). Il en résulte une charpente tridimensionnelle possédant des canaux larges parallèles repectivement aux deux directions [001] (Fig. 4) e t [-110] (Fig. 5) où se situent les cations monovalents Li^+^ et Na^+^. Les valeurs des charges des ions (BVS) dans la structure ont été calculées moyennant la formule empirique de Brown (Brown & Altermatt, 1985). Le résultat final: Mo1(6,23), Mo2(6,16), Mo3(6,21), Mo4(6,10), Mo5(6,20), Mo6(6,17), Na1(1,16), Na2(1,15), Na3(1,17), Na4(1,22) e t Li1(1,04) confirme bien le caractère haxavalent des atomes de molybden dans la phase étudiée. Une étude comparative de notre matériau avec des travaux antérieurs révèle un lien de parenté à celle de Na_6_Mo_11_O_36_ (Bramnik & Ehrenberg, 2004) qui peut être considéré comme un dérivé de la structure anatase. Ce pendant une différence nette est observée d'une part, dans la *sym*étrie des réseaux: l'anatase TiO_2_ (quatratique, groupe I41/amd), Na_6_Mo_11_O_36_ (triclinique, groupe P-1) e t notre composé Li_0.17_Na_5.83_Mo_11_O_36_ (monoclinique, groupe *C*2/*c*) et d'autre part dans la jonction des polyèdres dans la charpente. En effet, dans les deux composés Na_6_Mo_11_O_36_ et l'anatase TiO_2_ les octaèdres MO_6_ (*M*= Mo ou Ti) sont uniquement liés entre eux par des arêtes par contre dans la phase étudiée existent des pyramides MoO_5_ et la cohésion entre polyèdres est aussi renforsée par mize en commun de sommets.

### Experimental

Dans le but de substituer l'ion Na^+^ par Li^+^ dans Na_6_Mo_11_O_36_ (Bramnik & Ehrenberg, 2004), un mélange a été réalisé dans les rapports molaires Li:Na:Mo égaux à 1:5:11 à partir des réactifs solides LiNO_3_ (Fluka, 62575), NaCO_3_ (Fluka, 71350) e t (NH_4_)_2_Mo_4_O_13_ (Fluka, 69858). Il a été finement broyé et préchauffé à l'air à 573 K pendant une nuit. Après refroidissement et broyage, la préparation est portée, proche de la fusion pour favoraiser la germination et la croissance des cristaux, à 793 K pendant deux jours. le résidu final est refroidi lentement (5 K/jour) dans un intervalle de 50 degrés puis rapide jusqu'à la température ambiante. Par lavage à l'eau chaude des cristaux de couleur jaunâtre de qualité et de taille suffisante ont été séparés pour analyse par DRX.

### Refinement

*L*'examen des Facteurs de structure propose une *sym*étrie orthorombic groupe spacial Cccm, mais le facteur de concistence interne dans ce cas est *R*_int_= 27%. De plus, l'affinement dans ce système reste bloqué à 33% et les facteurs thermiques et les grandeurs géométriques sont mal définis. *L*'affinement a été conduit sans problème dans le système monoclinique (*R*_int_= 0,032) e t dans le groupe d'espace *C*2/*c*. A la fin de la résolution un examen de la Fourier-Différence finale révèle la présence d'un pic d'intensité faible situé à des distances interatomique des atomes d'oxygène correspondant bien au lithium mais ayant une agitation thermique très variable. *L*'utilization des fonctions SUMP et EADP, autorisées par le programme *SHELX*, pour les deux ions Na4 et Li1 conduit à des ellipsoïdes bien définis. De plus, Les densités d'électrons maximum et minimum restants dans la Fourier-différence sont acceptables et sont situées respectivements à 0,79 Å de Mo2 et à 0,91 Å de Mo1.

### Crystal data

**Table:**

Li_0.17_Na_5.83_Mo_11_O_36_	*F*(000) = 3259
*M**_r_* = 1766.55	*D*_x_ = 4.116 Mg m^−^^3^
Monoclinic, *C*2/*c*	Mo *K*α radiation, λ = 0.71073 Å
Hall symbol: -C 2yc	Cell parameters from 25 reflections
*a* = 7.2250 (9) Å	θ = 10–15°
*b* = 17.863 (2) Å	µ = 4.89 mm^−^^1^
*c* = 22.086 (3) Å	*T* = 298 K
β = 90.162 (8)°	Prism, yellow
*V* = 2850.5 (6) Å^3^	0.30 × 0.24 × 0.14 mm
*Z* = 4	

### Data collection

**Table:**

Enraf–Nonius CAD-4 diffractometer	3065 reflections with *I* > 2σ(*I*)
Radiation source: fine-focus sealed tube	*R*_int_ = 0.032
Graphite monochromator	θ_max_ = 27.2°, θ_min_ = 2.3°
ω/2θ scans	*h* = −9→9
Absorption correction: ψ scan (North *et al.*, 1968)	*k* = −1→22
*T*_min_ = 0.25, *T*_max_ = 0.50	*l* = −28→28
6662 measured reflections	2 standard reflections every 120 min
3138 independent reflections	intensity decay: 2.3%

### Refinement

**Table:**

Refinement on *F*^2^	Primary atom site location: structure-invariant direct methods
Least-squares matrix: full	Secondary atom site location: difference Fourier map
*R*[*F*^2^ > 2σ(*F*^2^)] = 0.037	*w* = 1/[σ^2^(*F*_o_^2^) + (0.0312*P*)^2^ + 53.4724*P*] where *P* = (*F*_o_^2^ + 2*F*_c_^2^)/3
*wR*(*F*^2^) = 0.095	(Δ/σ)_max_ < 0.001
*S* = 1.33	Δρ_max_ = 1.39 e Å^−^^3^
3138 reflections	Δρ_min_ = −1.85 e Å^−^^3^
247 parameters	Extinction correction: *SHELXL97* (Sheldrick, 2008), Fc^*^=kFc[1+0.001xFc^2^λ^3^/sin(2θ)]^-1/4^
1 restraint	Extinction coefficient: 0.00239 (8)

### Special details

**Table:**

Geometry. All e.s.d.'s (except the e.s.d. in the dihedral angle between two l.s. planes) are estimated using the full covariance matrix. The cell e.s.d.'s are taken into account individually in the estimation of e.s.d.'s in distances, angles and torsion angles; correlations between e.s.d.'s in cell parameters are only used when they are defined by crystal symmetry. An approximate (isotropic) treatment of cell e.s.d.'s is used for estimating e.s.d.'s involving l.s. planes.
Refinement. Refinement of *F*^2^ against ALL reflections. The weighted *R*-factor *wR* and goodness of fit *S* are based on *F*^2^, conventional *R*-factors *R* are based on *F*, with *F* set to zero for negative *F*^2^. The threshold expression of *F*^2^ > σ(*F*^2^) is used only for calculating *R*-factors(gt) *etc*. and is not relevant to the choice of reflections for refinement. *R*-factors based on *F*^2^ are statistically about twice as large as those based on *F*, and *R*- factors based on ALL data will be even larger.

### Fractional atomic coordinates and isotropic or equivalent isotropic displacement parameters (Å^2^)

**Table:**

	*x*	*y*	*z*	*U*_iso_*/*U*_eq_	Occ. (<1)
Mo1	−0.05004 (7)	0.09121 (3)	0.08276 (2)	0.00620 (14)	
Mo2	−0.04296 (7)	0.09488 (3)	−0.08670 (2)	0.00647 (15)	
Mo3	0.28669 (7)	0.16698 (3)	0.17148 (2)	0.00669 (15)	
Mo4	0.0000	0.06761 (4)	0.2500	0.00700 (18)	
Mo5	0.29608 (6)	0.15641 (3)	0.00098 (2)	0.00611 (14)	
Mo6	0.28716 (7)	0.15684 (3)	−0.17582 (2)	0.00792 (15)	
Na1	0.7353 (3)	0.24829 (16)	−0.17054 (12)	0.0162 (5)	
Na2	0.5000	−0.0049 (3)	0.2500	0.0223 (9)	
Na3	0.4865 (3)	−0.00129 (15)	0.08703 (11)	0.0146 (9)	0.974 (12)
Na4	0.7500	0.2500	0.0000	0.0144 (13)	0.884 (17)
Li1	0.0000	0.080 (5)	0.7500	0.0144 (13)	0.17 (3)
O1	0.8364 (6)	0.0093 (3)	0.91649 (19)	0.0096 (8)	
O2	0.1841 (6)	0.0087 (3)	0.2570 (2)	0.0155 (9)	
O3	0.0629 (6)	0.2560 (3)	0.8312 (2)	0.0114 (9)	
O4	0.8115 (6)	0.0125 (3)	0.0820 (2)	0.0116 (9)	
O5	0.4298 (6)	0.2375 (3)	0.0005 (2)	0.0116 (9)	
O6	0.4138 (5)	0.2410 (2)	0.84170 (19)	0.0085 (8)	
O7	0.0400 (6)	0.1004 (3)	0.83715 (19)	0.0105 (9)	
O8	0.7382 (7)	0.1602 (3)	0.7519 (2)	0.0183 (10)	
O9	0.2093 (6)	0.1534 (2)	0.9216 (2)	0.0093 (9)	
O10	0.0275 (6)	0.0968 (3)	0.16486 (18)	0.0097 (9)	
O11	0.2069 (6)	0.1538 (2)	0.08222 (19)	0.0086 (9)	
O12	0.0345 (6)	0.0951 (2)	0.00058 (18)	0.0098 (9)	
O13	0.4304 (7)	0.0913 (3)	0.1661 (2)	0.0152 (9)	
O14	0.2059 (7)	0.1611 (2)	0.2469 (2)	0.0111 (9)	
O15	0.7919 (6)	0.1631 (3)	0.9173 (2)	0.0145 (10)	
O16	0.4536 (6)	0.0861 (3)	0.0022 (2)	0.0145 (10)	
O17	0.7944 (7)	0.1625 (3)	0.0817 (2)	0.0157 (10)	
O18	0.4428 (7)	0.0853 (3)	0.8357 (2)	0.0139 (9)	

### Atomic displacement parameters (Å^2^)

**Table:**

	*U*^11^	*U*^22^	*U*^33^	*U*^12^	*U*^13^	*U*^23^
Mo1	0.0053 (2)	0.0088 (3)	0.0045 (2)	−0.00116 (18)	0.00002 (16)	0.00022 (17)
Mo2	0.0057 (2)	0.0083 (3)	0.0054 (2)	−0.00105 (17)	−0.00061 (17)	0.00018 (17)
Mo3	0.0053 (2)	0.0087 (3)	0.0060 (2)	−0.00102 (18)	−0.00057 (17)	0.00020 (17)
Mo4	0.0072 (3)	0.0091 (3)	0.0047 (3)	0.000	0.0014 (2)	0.000
Mo5	0.0057 (2)	0.0083 (3)	0.0044 (2)	−0.00079 (18)	0.00004 (17)	−0.00012 (17)
Mo6	0.0070 (2)	0.0083 (3)	0.0084 (3)	−0.00041 (18)	0.00160 (17)	0.00070 (17)
Na1	0.0115 (12)	0.0206 (14)	0.0166 (13)	−0.0003 (11)	−0.0004 (9)	−0.0012 (11)
Na2	0.0123 (17)	0.034 (2)	0.020 (2)	0.000	0.0043 (14)	0.000
Na3	0.0097 (13)	0.0197 (16)	0.0143 (15)	0.0014 (11)	−0.0013 (10)	0.0010 (11)
Na4	0.012 (2)	0.017 (2)	0.014 (2)	0.0019 (16)	−0.0013 (15)	−0.0008 (16)
Li1	0.012 (2)	0.017 (2)	0.014 (2)	0.0019 (16)	−0.0013 (15)	−0.0008 (16)
O1	0.0059 (18)	0.014 (2)	0.009 (2)	−0.0004 (17)	−0.0012 (14)	−0.0016 (16)
O2	0.013 (2)	0.015 (2)	0.018 (2)	0.0023 (19)	0.0045 (17)	0.0058 (19)
O3	0.0074 (19)	0.012 (2)	0.015 (2)	−0.0013 (17)	−0.0008 (16)	0.0025 (17)
O4	0.0072 (19)	0.015 (2)	0.012 (2)	−0.0031 (17)	0.0005 (16)	−0.0022 (17)
O5	0.0109 (19)	0.013 (2)	0.011 (2)	−0.0021 (18)	−0.0003 (16)	0.0008 (17)
O6	0.0052 (17)	0.010 (2)	0.010 (2)	−0.0023 (16)	0.0013 (15)	−0.0002 (16)
O7	0.012 (2)	0.012 (2)	0.008 (2)	−0.0028 (17)	0.0002 (15)	−0.0005 (16)
O8	0.021 (3)	0.022 (3)	0.012 (2)	0.005 (2)	−0.0009 (18)	−0.0007 (19)
O9	0.011 (2)	0.011 (2)	0.0065 (19)	−0.0018 (16)	0.0010 (16)	−0.0036 (15)
O10	0.011 (2)	0.015 (2)	0.0028 (19)	−0.0045 (17)	0.0008 (15)	0.0009 (15)
O11	0.009 (2)	0.011 (2)	0.0052 (19)	−0.0031 (16)	0.0013 (15)	−0.0009 (15)
O12	0.013 (2)	0.014 (2)	0.0029 (18)	−0.0053 (17)	0.0017 (16)	−0.0010 (15)
O13	0.016 (2)	0.014 (2)	0.015 (2)	0.0051 (18)	−0.0008 (17)	0.0000 (18)
O14	0.014 (2)	0.014 (2)	0.0057 (19)	−0.0003 (17)	0.0002 (16)	−0.0009 (16)
O15	0.014 (2)	0.015 (2)	0.014 (2)	0.0034 (18)	0.0010 (18)	0.0006 (17)
O16	0.016 (2)	0.012 (2)	0.015 (2)	0.0039 (18)	−0.0007 (18)	−0.0012 (18)
O17	0.017 (2)	0.015 (2)	0.014 (2)	0.0004 (18)	−0.0015 (18)	0.0026 (18)
O18	0.015 (2)	0.012 (2)	0.014 (2)	0.0038 (18)	0.0031 (17)	0.0010 (17)

### Geometric parameters (Å, º)

**Table:**

Mo1—O17^i^	1.699 (5)	Na3—O1^ii^	2.338 (5)
Mo1—O4^i^	1.725 (4)	Na3—O4	2.364 (5)
Mo1—O10	1.899 (4)	Na3—O13	2.440 (5)
Mo1—O12	1.918 (4)	Na3—O16	2.449 (5)
Mo1—O11	2.167 (4)	Na3—O16^xiii^	2.525 (5)
Mo1—O1^ii^	2.368 (4)	Na4—O5^xi^	2.324 (4)
Mo2—O15^iii^	1.708 (5)	Na4—O5	2.324 (4)
Mo2—O1^iii^	1.761 (4)	Na4—O17^xi^	2.408 (5)
Mo2—O7^iv^	1.790 (4)	Na4—O17	2.408 (5)
Mo2—O12	2.006 (4)	Na4—O15^xiv^	2.417 (5)
Mo2—O9^iv^	2.109 (4)	Na4—O15^iv^	2.417 (5)
Mo3—O13	1.709 (5)	Li1—O7	1.979 (18)
Mo3—O3^v^	1.755 (4)	Li1—O7^xv^	1.979 (18)
Mo3—O14	1.769 (5)	Li1—O2^xvi^	2.08 (8)
Mo3—O11	2.066 (4)	Li1—O2^xvii^	2.08 (8)
Mo3—O6^v^	2.210 (4)	Li1—O8^xviii^	2.37 (6)
Mo3—O10	2.258 (4)	Li1—O8^i^	2.37 (6)
Mo4—O2^vi^	1.703 (5)	O1—Mo2^xix^	1.761 (4)
Mo4—O2	1.703 (5)	O1—Na3^ii^	2.338 (5)
Mo4—O10^vi^	1.962 (4)	O1—Mo1^ii^	2.368 (4)
Mo4—O10	1.962 (4)	O2—Li1^xvi^	2.08 (8)
Mo4—O14	2.238 (5)	O3—Mo3^v^	1.755 (4)
Mo4—O14^vi^	2.238 (5)	O3—Na1^xx^	2.371 (5)
Mo5—O16	1.695 (5)	O3—Mo6^xxi^	2.405 (4)
Mo5—O5	1.741 (4)	O4—Mo1^xxii^	1.725 (4)
Mo5—O9^iv^	1.862 (4)	O5—Mo5^vii^	2.501 (5)
Mo5—O11	1.909 (4)	O6—Mo6^xxi^	1.802 (4)
Mo5—O12	2.184 (4)	O6—Mo3^v^	2.210 (4)
Mo5—O5^vii^	2.501 (5)	O6—Na1^xxi^	2.342 (5)
Mo6—O8^viii^	1.690 (5)	O7—Mo2^xxi^	1.790 (4)
Mo6—O18^iv^	1.720 (5)	O7—Mo6^xxi^	2.072 (5)
Mo6—O6^iv^	1.802 (4)	O8—Mo6^viii^	1.690 (5)
Mo6—O7^iv^	2.072 (5)	O8—Na1^xxi^	2.327 (6)
Mo6—O9^iv^	2.225 (4)	O8—Li1^xxii^	2.37 (6)
Mo6—O3^iv^	2.405 (4)	O9—Mo5^xxi^	1.862 (4)
Na1—O8^iv^	2.327 (6)	O9—Mo2^xxi^	2.109 (4)
Na1—O6^iv^	2.342 (5)	O9—Mo6^xxi^	2.225 (4)
Na1—O3^ix^	2.371 (5)	O14—Na1^xxiii^	2.448 (5)
Na1—O14^x^	2.448 (5)	O15—Mo2^xix^	1.708 (5)
Na1—O15^iv^	2.499 (5)	O15—Na4^xxi^	2.417 (5)
Na1—O17^xi^	2.537 (6)	O15—Na1^xxi^	2.499 (5)
Na2—O2^viii^	2.301 (5)	O16—Na3^xiii^	2.525 (5)
Na2—O2	2.301 (5)	O17—Mo1^xxii^	1.699 (5)
Na2—O18^ii^	2.413 (5)	O17—Na1^xi^	2.537 (6)
Na2—O18^xii^	2.413 (5)	O18—Mo6^xxi^	1.720 (5)
Na2—O13^viii^	2.575 (6)	O18—Na3^ii^	2.329 (5)
Na2—O13	2.575 (6)	O18—Na2^ii^	2.413 (5)
Na3—O18^ii^	2.329 (5)		
			
O17^i^—Mo1—O4^i^	103.1 (2)	O8^viii^—Mo6—O3^iv^	88.1 (2)
O17^i^—Mo1—O10	99.6 (2)	O18^iv^—Mo6—O3^iv^	167.72 (19)
O4^i^—Mo1—O10	102.8 (2)	O6^iv^—Mo6—O3^iv^	73.36 (17)
O17^i^—Mo1—O12	99.9 (2)	O7^iv^—Mo6—O3^iv^	76.61 (16)
O4^i^—Mo1—O12	101.9 (2)	O9^iv^—Mo6—O3^iv^	77.62 (16)
O10—Mo1—O12	143.9 (2)	O8^iv^—Na1—O6^iv^	93.37 (19)
O17^i^—Mo1—O11	100.4 (2)	O8^iv^—Na1—O3^ix^	92.29 (19)
O4^i^—Mo1—O11	156.48 (19)	O6^iv^—Na1—O3^ix^	172.4 (2)
O10—Mo1—O11	74.20 (17)	O8^iv^—Na1—O14^x^	84.20 (18)
O12—Mo1—O11	72.60 (17)	O6^iv^—Na1—O14^x^	92.23 (18)
O17^i^—Mo1—O1^ii^	179.2 (2)	O3^ix^—Na1—O14^x^	93.35 (18)
O4^i^—Mo1—O1^ii^	76.14 (18)	O8^iv^—Na1—O15^iv^	99.12 (19)
O10—Mo1—O1^ii^	80.95 (17)	O6^iv^—Na1—O15^iv^	92.11 (18)
O12—Mo1—O1^ii^	79.90 (17)	O3^ix^—Na1—O15^iv^	82.02 (17)
O11—Mo1—O1^ii^	80.36 (16)	O14^x^—Na1—O15^iv^	174.4 (2)
O15^iii^—Mo2—O1^iii^	105.7 (2)	O8^iv^—Na1—O17^xi^	174.5 (2)
O15^iii^—Mo2—O7^iv^	104.2 (2)	O6^iv^—Na1—O17^xi^	81.93 (17)
O1^iii^—Mo2—O7^iv^	104.6 (2)	O3^ix^—Na1—O17^xi^	92.15 (18)
O15^iii^—Mo2—O12	98.1 (2)	O14^x^—Na1—O17^xi^	98.84 (18)
O1^iii^—Mo2—O12	95.73 (19)	O15^iv^—Na1—O17^xi^	78.23 (17)
O7^iv^—Mo2—O12	144.1 (2)	O2^viii^—Na2—O2	167.9 (3)
O15^iii^—Mo2—O9^iv^	104.2 (2)	O2^viii^—Na2—O18^ii^	80.68 (17)
O1^iii^—Mo2—O9^iv^	148.71 (18)	O2—Na2—O18^ii^	106.73 (19)
O7^iv^—Mo2—O9^iv^	76.26 (18)	O2^viii^—Na2—O18^xii^	106.73 (19)
O12—Mo2—O9^iv^	71.17 (17)	O2—Na2—O18^xii^	80.68 (17)
O13—Mo3—O3^v^	104.0 (2)	O18^ii^—Na2—O18^xii^	106.9 (3)
O13—Mo3—O14	102.7 (2)	O2^viii^—Na2—O13^viii^	77.65 (17)
O3^v^—Mo3—O14	106.5 (2)	O2—Na2—O13^viii^	94.17 (19)
O13—Mo3—O11	90.7 (2)	O18^ii^—Na2—O13^viii^	158.13 (16)
O3^v^—Mo3—O11	103.19 (19)	O18^xii^—Na2—O13^viii^	82.33 (15)
O14—Mo3—O11	143.1 (2)	O2^viii^—Na2—O13	94.17 (19)
O13—Mo3—O6^v^	167.8 (2)	O2—Na2—O13	77.65 (17)
O3^v^—Mo3—O6^v^	79.52 (18)	O18^ii^—Na2—O13	82.33 (15)
O14—Mo3—O6^v^	87.15 (19)	O18^xii^—Na2—O13	158.13 (16)
O11—Mo3—O6^v^	77.19 (16)	O13^viii^—Na2—O13	96.3 (3)
O13—Mo3—O10	93.5 (2)	O18^ii^—Na3—O1^ii^	101.64 (19)
O3^v^—Mo3—O10	161.23 (18)	O18^ii^—Na3—O4	83.45 (18)
O14—Mo3—O10	75.65 (18)	O1^ii^—Na3—O4	174.76 (19)
O11—Mo3—O10	69.26 (15)	O18^ii^—Na3—O13	87.07 (18)
O6^v^—Mo3—O10	82.01 (16)	O1^ii^—Na3—O13	84.13 (18)
O2^vi^—Mo4—O2	103.6 (3)	O4—Na3—O13	97.46 (19)
O2^vi^—Mo4—O10^vi^	99.8 (2)	O18^ii^—Na3—O16	172.73 (19)
O2—Mo4—O10^vi^	99.1 (2)	O1^ii^—Na3—O16	85.35 (17)
O2^vi^—Mo4—O10	99.1 (2)	O4—Na3—O16	89.52 (18)
O2—Mo4—O10	99.8 (2)	O13—Na3—O16	95.73 (18)
O10^vi^—Mo4—O10	149.2 (3)	O18^ii^—Na3—O16^xiii^	98.48 (18)
O2^vi^—Mo4—O14	167.9 (2)	O1^ii^—Na3—O16^xiii^	96.35 (17)
O2—Mo4—O14	86.8 (2)	O4—Na3—O16^xiii^	81.56 (17)
O10^vi^—Mo4—O14	84.27 (18)	O13—Na3—O16^xiii^	174.19 (19)
O10—Mo4—O14	72.72 (17)	O16—Na3—O16^xiii^	78.56 (18)
O2^vi^—Mo4—O14^vi^	86.8 (2)	O5^xi^—Na4—O5	180.0
O2—Mo4—O14^vi^	167.9 (2)	O5^xi^—Na4—O17^xi^	93.72 (16)
O10^vi^—Mo4—O14^vi^	72.72 (17)	O5—Na4—O17^xi^	86.28 (16)
O10—Mo4—O14^vi^	84.27 (18)	O5^xi^—Na4—O17	86.28 (16)
O14—Mo4—O14^vi^	83.5 (2)	O5—Na4—O17	93.72 (16)
O16—Mo5—O5	104.1 (2)	O17^xi^—Na4—O17	180.0 (2)
O16—Mo5—O9^iv^	102.6 (2)	O5^xi^—Na4—O15^xiv^	93.92 (16)
O5—Mo5—O9^iv^	101.7 (2)	O5—Na4—O15^xiv^	86.08 (16)
O16—Mo5—O11	101.2 (2)	O17^xi^—Na4—O15^xiv^	97.62 (16)
O5—Mo5—O11	102.4 (2)	O17—Na4—O15^xiv^	82.38 (16)
O9^iv^—Mo5—O11	140.48 (19)	O5^xi^—Na4—O15^iv^	86.08 (16)
O16—Mo5—O12	102.1 (2)	O5—Na4—O15^iv^	93.92 (16)
O5—Mo5—O12	153.79 (19)	O17^xi^—Na4—O15^iv^	82.38 (16)
O9^iv^—Mo5—O12	72.12 (17)	O17—Na4—O15^iv^	97.62 (16)
O11—Mo5—O12	72.37 (17)	O15^xiv^—Na4—O15^iv^	180.0 (2)
O16—Mo5—O5^vii^	178.54 (19)	O7—Li1—O7^xv^	159 (6)
O5—Mo5—O5^vii^	74.42 (19)	O7—Li1—O2^xvi^	108 (2)
O9^iv^—Mo5—O5^vii^	77.99 (17)	O7^xv^—Li1—O2^xvi^	88.6 (17)
O11—Mo5—O5^vii^	78.98 (17)	O7—Li1—O2^xvii^	88.6 (17)
O12—Mo5—O5^vii^	79.37 (15)	O7^xv^—Li1—O2^xvii^	108 (2)
O8^viii^—Mo6—O18^iv^	104.1 (2)	O2^xvi^—Li1—O2^xvii^	80 (3)
O8^viii^—Mo6—O6^iv^	103.7 (2)	O7—Li1—O8^xviii^	78.0 (18)
O18^iv^—Mo6—O6^iv^	104.9 (2)	O7^xv^—Li1—O8^xviii^	89 (2)
O8^viii^—Mo6—O7^iv^	93.7 (2)	O2^xvi^—Li1—O8^xviii^	166 (3)
O18^iv^—Mo6—O7^iv^	100.4 (2)	O2^xvii^—Li1—O8^xviii^	87.2 (2)
O6^iv^—Mo6—O7^iv^	144.52 (18)	O7—Li1—O8^i^	89 (2)
O8^viii^—Mo6—O9^iv^	159.1 (2)	O7^xv^—Li1—O8^i^	78.0 (18)
O18^iv^—Mo6—O9^iv^	90.19 (19)	O2^xvi^—Li1—O8^i^	87.2 (2)
O6^iv^—Mo6—O9^iv^	86.85 (17)	O2^xvii^—Li1—O8^i^	166 (3)
O7^iv^—Mo6—O9^iv^	68.45 (16)	O8^xviii^—Li1—O8^i^	106 (4)

Symmetry codes: (i) *x*−1, *y*, *z*; (ii) −*x*+1, −*y*, −*z*+1; (iii) *x*−1, *y*, *z*−1; (iv) *x*, *y*, *z*−1; (v) −*x*+1/2, −*y*+1/2, −*z*+1; (vi) −*x*, *y*, −*z*+1/2; (vii) −*x*+1/2, −*y*+1/2, −*z*; (viii) −*x*+1, *y*, −*z*+1/2; (ix) *x*+1, *y*, *z*−1; (x) *x*+1/2, −*y*+1/2, *z*−1/2; (xi) −*x*+3/2, −*y*+1/2, −*z*; (xii) *x*, −*y*, *z*−1/2; (xiii) −*x*+1, −*y*, −*z*; (xiv) −*x*+3/2, −*y*+1/2, −*z*+1; (xv) −*x*, *y*, −*z*+3/2; (xvi) −*x*, −*y*, −*z*+1; (xvii) *x*, −*y*, *z*+1/2; (xviii) −*x*+1, *y*, −*z*+3/2; (xix) *x*+1, *y*, *z*+1; (xx) *x*−1, *y*, *z*+1; (xxi) *x*, *y*, *z*+1; (xxii) *x*+1, *y*, *z*; (xxiii) *x*−1/2, −*y*+1/2, *z*+1/2.
